# Cutaneous mantle cell lymphoma presenting as a diffuse morbilliform rash: A case report

**DOI:** 10.1177/2050313X231212994

**Published:** 2023-11-17

**Authors:** Spencer Tuohy, Christopher Wachuku, Yixin A Wang, Aman Prasad, Sara S Samimi, Lisa K Pappas-Taffer, Paul L Haun, Leo L Wang

**Affiliations:** Department of Dermatology, Perelman School of Medicine, University of Pennsylvania, Philadelphia, PA, USA

**Keywords:** Cancer, dermatology

## Abstract

This case describes a patient with known mantle cell lymphoma without cutaneous involvement presenting with a diffuse morbilliform rash during an inpatient admission for bacterial pneumonia. The patient was thought to have a hypersensitivity to antibiotics but failed to improve after the offending agents were stopped. A skin biopsy revealed metastatic cutaneous mantle cell lymphoma. Treatment with high-dose corticosteroids and chemotherapy was initiated resulting in the resolution of the rash.

## Introduction

Mantle cell lymphoma (MCL) is a rare, aggressive malignancy that represents 3%–6% of non-Hodgkin’s lymphoma.^1^ Cutaneous metastases in MCL are even rarer and found only in 1.4% of MCL patients.^2^ When present, cutaneous disease typically presents locally as dermal tumors.^2^ Here, we describe a case of diffuse morbilliform cutaneous MCL.

## Case report

A male in his 70s with known MCL on zanubrutinib presented to the medical intensive care unit with hypoxic respiratory failure from bacterial pneumonia. His medical history was notable for nonmelanoma skin cancers, carotid artery stenosis, hyperlipidemia, and myotonic dystrophy. He was treated initially with cefepime and azithromycin for bacterial pneumonia. Due to his history of immunosuppression, trimethoprim–sulfamethoxazole was started with adjuvant prednisone of 80 mg/day for *Pneumocystis jiroveci* pneumonia.

**Figure 1.**
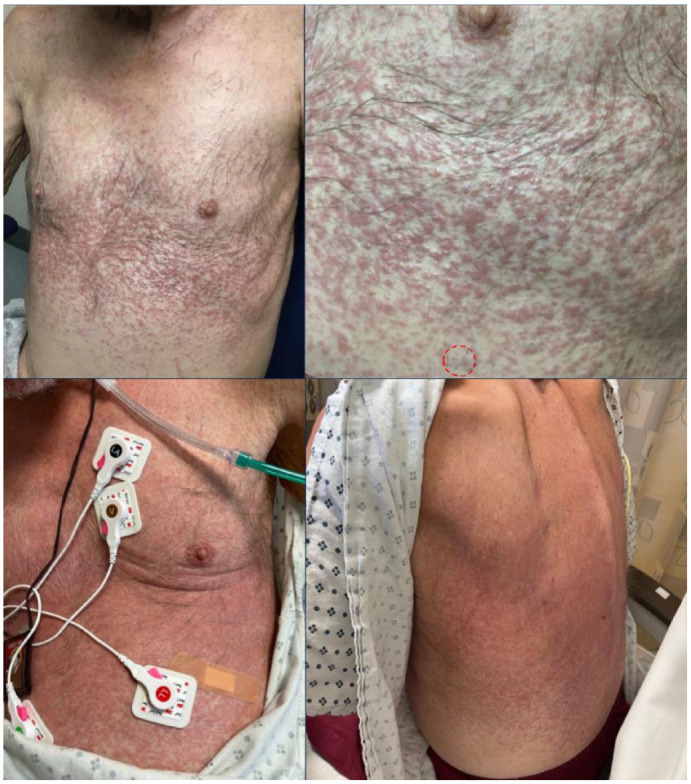
Clinical presentation of mantle cell lymphoma. Top row: Mantle cell lymphoma presenting as an edematous diffuse morbilliform rash, biopsy site highlighted in the second panel in red. Bottom row: similar presentation and minimal improvement after withdrawal of offending medications and one week of topical steroids.

One week later, he developed a diffuse morbilliform rash starting from his trunk in the setting after his prednisone dose of 20 mg/day. On examination, he was noted to have edematous pink macules and papules on his trunk and extremities, most prominent over his lower chest and back (Figure 1, top row). His laboratory results were notable for a white blood cell count of 216k/µL with marked eosinophilia, 8800/µL (4%). A complete metabolic panel was unremarkable. At this time, he was treated for a suspected hypersensitivity to cefepime, azithromycin, or trimethoprim–sulfamethaxazole, which were promptly stopped. He was started on topical triamcinolone ointment twice daily with minimal improvement after 1 week (Figure 1, bottom row).

**Figure 2.**
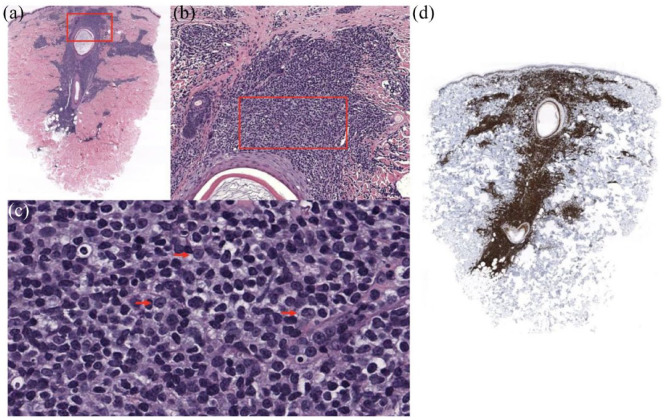
Punch biopsy of the left lower abdomen showing (a) dense dermal infiltrate surrounding the follicle; (b) atypical monomorphic lymphocytes; (c) ovoid nuclei and open and dispersed chromatin (highlighted by red arrows); and (d) positive cyclin D1 staining.

A punch biopsy was obtained which demonstrated atypical monomorphic lymphocytes with ovoid nuclei and open and dispersed chromatin (Figure 2). Chromatin stains were positive for CD20, Cyclin D1, and PAX5, consistent with a diagnosis of cutaneous MCL. Prednisone was increased to 100 mg/day resulting in improvement in rash, leukocytosis, and eosinophilia. Due to the progression of his disease, cytoreduction with bendamustine was initiated.

## Discussion

MCL is a B-cell lymphoma with a nonspecific clinical presentation, including lymphadenopathy and extranodal involvement at sites including the peripheral blood, bone marrow, and gastrointestinal tract.^1^ Around 80% of MCL patients are diagnosed at stage III or IV.^2^ MCL has a characteristic translocation of *t*(11,14)(q13,q32) joining the IGH (Immunoglobulin Heavy Locus) and CCND1 (cyclin D1) genes in the mantle zone. This leads to the overexpression of cell-cycle regulator cyclin D1. The typical markers for MCL include CD19, CD20, CD22, CD79a, and CD5 which alongside cyclin D1 overexpression lead to the diagnosis of MCL.^3^

Cutaneous mantle cell lymphoma is exceptionally rare and typically presents as infiltrated dermal tumors.^2,4,5^ In a case series of 10 patients with cutaneous MCL confirmed on histology, all patients presented in the form of nodules or tumors.^4^ Another case series identified 18 patients representing 6 nodular lesions, 6 localized macular or maculopapular lesions, 4 infiltrated plaques, and 2 subcutaneous nodules.^3^ One case reported a diffuse petechial eruption of cutaneous mantle cell lymphoma, which was most similar to this clinical presentation.^6^

In our case, there was a clinical concern for hypersensitivity to trimethoprim–sulfamethoxazole or cefepime. Clinically, this was the most likely etiology for the patient’s rash given the nonspecific presentation. We further hypothesized that lymphoma cells may be recruited to the skin due to drug hypersensitivity. However, the rash had an atypical distribution; it started first on the upper abdomen and mid-back, spreading slowly to the extremities. Furthermore, the rash failed to improve after antibiotic withdrawal and consistent use of topical steroids. Finally, the lymphoma cell pattern favored a metastatic pattern on histology with a diffuse infiltrate around the adnexa compared to a reactive one in which we would expect a perivascular distribution. Together, this was suggestive of metastatic cutaneous MCL, an underrecognized pattern of cutaneous lymphoma.

This case underscores the importance of understanding the heterogeneous presentation of cutaneous MCL and the need to include a broad differential for rashes in patients with known hematologic malignancies. Clinicians should be aware that atypical morphologies and distribution should raise suspicion for a metastatic process. In areas of diagnostic uncertainty, a biopsy is critical, as cutaneous hematologic malignancies such as cutaneous MCL are managed aggressively with high doses of corticosteroids, radiation, and chemotherapy.

## Conclusions

Cutaneous MCL is exceptionally rare and when present typically presents in a single discrete area. This study raises awareness of a diffuse morbilliform eruption of cutaneous MCL that could easily be mistaken as a drug hypersensitivity. Recognition and biopsy of suspicious morbilliform eruptions can impact treatment and patient outcomes.
